# Evaluation of mutation rates, mosaicism and off target mutations when injecting Cas9 mRNA or protein for genome editing of bovine embryos

**DOI:** 10.1038/s41598-020-78264-8

**Published:** 2020-12-18

**Authors:** Sadie L. Hennig, Joseph R. Owen, Jason C. Lin, Amy E. Young, Pablo J. Ross, Alison L. Van Eenennaam, James D. Murray

**Affiliations:** 1 Department of Animal Science, University of California – Davis, Davis, CA USA; 2 Department of Population Health and Reproduction, University of California – Davis, Davis, CA USA

**Keywords:** Biological techniques, Biotechnology, Genetics

## Abstract

The CRISPR/Cas9 genome editing tool has the potential to improve the livestock breeding industry by allowing for the introduction of desirable traits. Although an efficient and targeted tool, the CRISPR/Cas9 system can have some drawbacks, including off-target mutations and mosaicism, particularly when used in developing embryos. Here, we introduced genome editing reagents into single-cell bovine embryos to compare the effect of Cas9 mRNA and protein on the mutation efficiency, level of mosaicism, and evaluate potential off-target mutations utilizing next generation sequencing. We designed guide-RNAs targeting three loci (POLLED, H11, and ZFX) in the bovine genome and saw a significantly higher rate of mutation in embryos injected with Cas9 protein (84.2%) vs. Cas9 mRNA (68.5%). In addition, the level of mosaicism was higher in embryos injected with Cas9 mRNA (100%) compared to those injected with Cas9 protein (94.2%), with little to no unintended off-target mutations detected. This study demonstrated that the use of gRNA/Cas9 ribonucleoprotein complex resulted in a high editing efficiency at three different loci in bovine embryos and decreased levels of mosaicism relative to Cas9 mRNA. Additional optimization will be required to further reduce mosaicism to levels that make single-step embryo editing in cattle commercially feasible.

## Introduction

CRISPR-mediated genome editing in livestock zygotes offers an attractive approach to introduce useful genetic variation into the next generation of cattle breeding programs. However, genetic mosaicism is particularly problematic for CRISPR-mediated genome editing in developing zygotes^1,2^. Genetic mosaicism complicates phenotypic analysis of F0 animals and may complicate screening multiple founders and breeding mosaic founders to produce an F1 generation. While this is routine in plant and mouse research, such approaches are time-consuming and essentially cost-prohibitive in uniparous large food animal species with long generation intervals like cattle.

A limited number of genome editing studies have been reported in bovine zygotes^3^, and indicate the frequent production of mosaic embryos. The frequency of mosaicism varies depending upon the type of site-directed nuclease used, the timing of editing relative to embryonic development, the form and efficiency of the targeting regents, the intrinsic properties of the target locus, and the method of delivery^1^.

Correspondingly, there are a number of experimental variables that need to be optimized to improve the efficiency of obtaining non-mosaic, homozygous genome edited founder cattle. In this study, we focused on the type of CRISPR/Cas9 system delivered (i.e. mRNA or protein) and report the impact on mutation efficiency, levels of mosaicism, and off-target mutations based on next generation sequencing when using CRISPR-mediated genome editing of bovine zygotes.

## Results

### Guide construction and testing

To determine the optimal parameters for CRISPR/Cas9-mediated genome editing in bovine zygotes, efficiency following microinjection was investigated for three gRNA per locus on three different chromosomes. Three gRNAs were designed targeting the POLLED locus on chromosome 1, a safe harbor locus (H11) on chromosome 17 and a locus (ZFX) on the X-chromosome downstream of the Zinc Finger, X-linked gene (Supplementary Table S1). Three gRNAs per locus were independently injected alongside Cas9 protein in groups of 30 zygotes, 18 h post insemination (hpi). Groups of 50 non-injected embryos were cultured as controls. The highest mutation rates were 76.9% for gRNA2 targeting the POLLED locus, 83.3% for gRNA1 targeting the H11 locus, and 77.8% for gRNA3 targeting the ZFX locus (Supplementary Table S2; χ^2^ test, *P* < 0.05). Overall, there was a decrease in the number of embryos that reached the blastocyst stage as the rate of mutation for a given gRNA increased. For each locus, the gRNA with the highest mutation rate was associated with the lowest developmental rate (Supplementary Table S2). gRNAs with the highest mutation rate were selected for further analysis.

**Figure 1:**
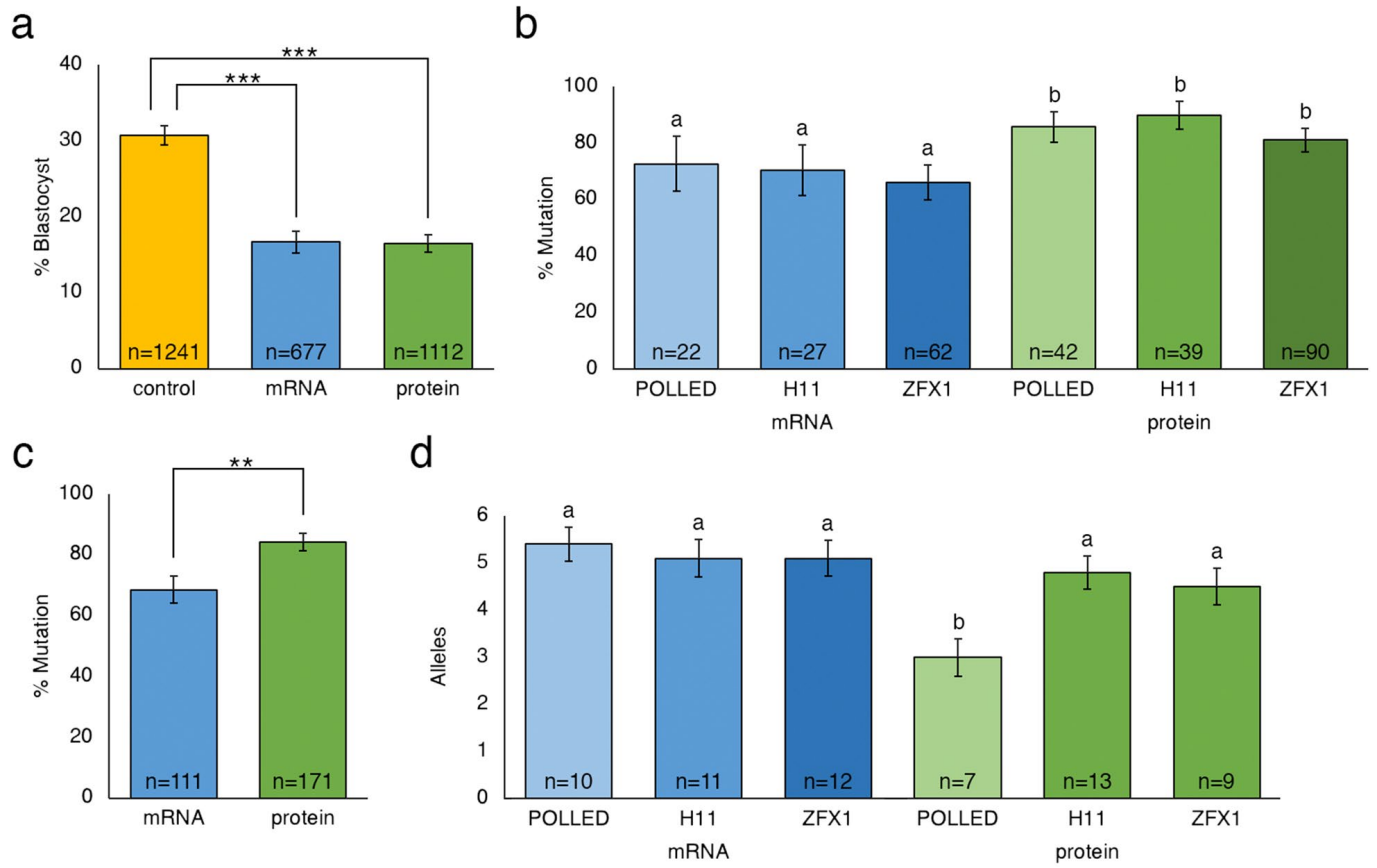
Percentage of uninjected control and microinjected zygotes reaching the blastocyst developmental stage following microinjection of either Cas9 mRNA or protein into in vitro fertilized bovine embryos 18 h post insemination, and percentage analyzed blastocysts with Cas9-induced mutations. (**a**) Blastocyst developmental percentage of CRISPR injected zygotes for all three loci compared to control non-injected zygotes. (**b**) Percentage of blastocysts with Cas9 mRNA or protein-induced mutation by all gRNAs targeting three loci (POLLED, H11, and ZFX) in the bovine genome. (**c**) Percentage of blastocysts with Cas9-induced mutations when injecting either Cas9 mRNA or protein alongside gRNAs targeting all three loci. (**d**) Average number of alleles per blastocyst when injecting Cas9 mRNA or protein targeting three loci (POLLED, H11, and ZFX) in the bovine genome. Error bars = standard error of the mean. **P < 0.005; ***P < 0.0005. Columns with differing letters in the same graph are significantly different (*P* < 0.05).

Guides targeting the POLLED locus, the H11 locus and the ZFX locus were then injected in groups of 30 in vitro fertilized embryos 18hpi alongside either Cas9 mRNA or protein (Table 1). The blastocyst rate of uninjected controls (30.7%) was significantly higher than embryos that were microinjected with gRNA and Cas9 editing reagents (Fig. 1a; *P* < 0.001). The overall mutation rate did not differ among the three loci for a given form of Cas9 (Fig. 1b; *P* = 0.45;); however the probability of a mutation was higher (*P* = 0.002) when Cas9 protein was microinjected as compared to Cas9 mRNA (Fig. 1c).

**Table 1:** Number of zygotes reaching the blastocyst developmental stage following microinjection of either Cas9 mRNA or protein and gRNAs targeting three loci (POLLED, H11, and ZFX) on different chromosomes. In vitro fertilized bovine embryos were injected 18 h post insemination, and the percentage of blastocysts with Cas9-induced mutations was determined by sequence analysis. Letters that differ in the same column are significantly different (*P* < 0.01).

Cas9	gRNA	Injected groups	Total embryos	Total blasts (%)	Total analyzed	Total mutation (%)
mRNA	Control	–	492	131 (27)^a^	–	–
	POLLED	4	114	22 (19)^b^	22	16 (73)^a^
	H11	7	191	28 (15)^b^	27	19 (70)^a^
	ZFX	14	372	63 (16)^b^	62	41 (67)^a^
protein	Control	–	749	250 (33)^a^	–	–
	POLLED	12	316	53 (17)^b^	42	36 (86)^b^
	H11	8	234	39 (17)^b^	39	35 (90)^b^
	ZFX	22	562	91 (16)^b^	90	73 (81)^b^

### Evaluation of mosaicism and off-target insertions and deletions

To evaluate the level of mosaicism, 69 blastocysts (19 gRNA2 targeting the POLLED locus (10 Cas9 mRNA, 9 Cas9 protein), 26 gRNA1 targeting the H11 locus (11 Cas9 mRNA, 15 Cas9 protein), and 24 targeting the ZFX locus (13 Cas9 mRNA, 11 Cas9 protein)) were collected, barcoded by PCR amplification and sequenced on a PacBio sequencer (Supplementary Table S3). Consensus sequences were called from raw reads using circular consensus sequencing (ccs) with a minimum of 3 passes, a minimum predicted accuracy of 99% and a maximum length of 700 bp (Supplementary Table S4). Unsorted ccs reads were aligned to each of the target sequences to analyze the types of insertions/deletions (indels) surrounding the predicted cut site with 26,460 reads aligned to the POLLED target site; 78,305 reads aligned to the H11 target site; and 66,780 reads aligned to ZFX target site (Supplementary Table S5). About half of the aligned sequences for the POLLED locus were wild type sequences (47.8%), while almost three quarters of the H11 and ZFX reads were wild type sequences (75.7% and 71.3%, respectively). The primary indels for reads aligned to the POLLED locus were 7 bp deletion (1672 reads), 11 bp deletion (1751), 4 bp deletion (6356 reads) and 1 bp insertion (2250 reads); aligned to the H11 locus were 11 bp deletion (3246 reads), 6 bp deletion (3813 reads), 3 bp deletion (4091 reads), and 1 bp deletion (7853 reads); and aligned to the ZFX locus were 14 bp deletion (4222 reads), 9 bp deletion (2998 reads), 3 bp deletion (3198 reads), 1 bp deletion (2194 reads) and 1 bp insertion (6532 reads) (Supplementary Table S5).

**Figure 2:**
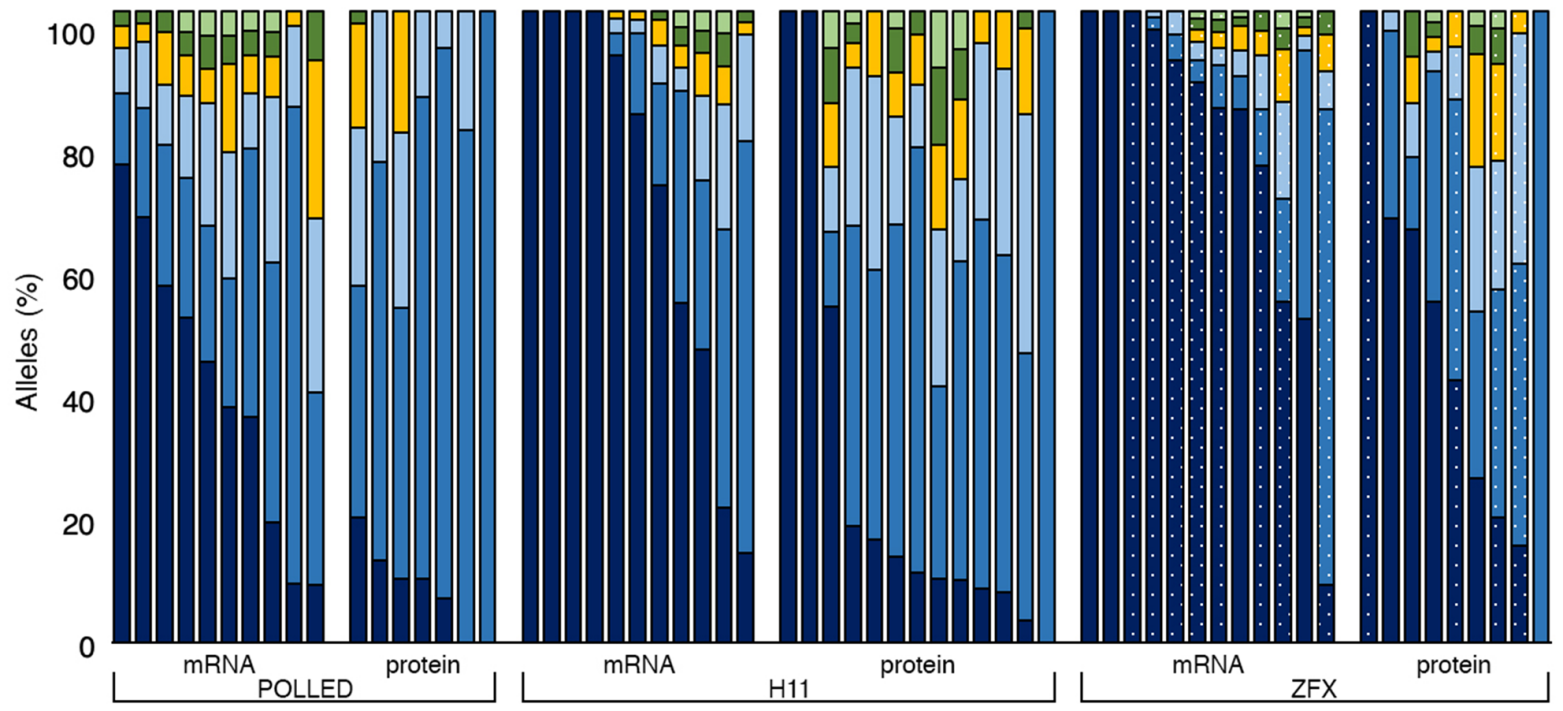
Bar graph depicting the percentage of alleles determined by PacBio sequencing in each of the 62 blastocysts microinjected 18 h post insemination with either Cas9 mRNA or protein and gRNAs targeting the POLLED, H11 and ZFX loci. Samples contained some combination of the wild type allele (dark blue) or an allele containing an insertion or deletion mediated by non-homologous end joining (blue, light blue, yellow, dark green and light green). For ZFX locus: dotted bars are female; solid bars are male.

Ccs reads were then sorted by barcode and analyzed by individual embryos (Fig. 2). Seven samples were discarded from further analysis due to a lack of reads following the quality filtering step (Supplemental Table S3). A total of 10 samples contained only wild type sequence (7 Cas9 mRNA and 3 Cas9 protein), resulting in an overall mutation rate of ~ 84% (Table 2). Of the 62 samples injected 18hpi, four contained only mutated alleles, without evidence for any wild type sequence. All four samples were from embryos injected with Cas9 protein (Supplementary Table S6). Three of these samples contained only one allele and were presumably non-mosaic homozygous, although our analyses could not rule out an unmappable mutation (e.g. large insertion) at the second allele. Each of the mutated embryos containing more than a single allele had at least three individual alleles or a disproportion of reads for each allele, for example 75% wildtype and 25% mutant (Supplementary Fig. S1), suggesting these embryos were mosaic rather than heterozygous. This translates to 94.2% mosaicism when injecting Cas9 protein compared to 100% mosaicism when injecting Cas9 mRNA.

**Table 2:** Editing efficiencies, mosaicism, average number of alleles and percent wild type reads as determined by PacBio sequencing of 63 blastocysts following microinjection of Cas9 mRNA or protein alongside gRNAs targeting three loci (POLLED, H11, and ZFX) on different chromosomes. I*n vitro* fertilized bovine embryos were injected 18 h post insemination. Letters that differ in the same column are significantly different (P < 0.05). SEM = standard error of the mean.

Locus	n	Cas9	% non-edited	% edited non-mosaic	% mosaic embryos	Alleles	SEM	% Wild type	SEM
POLLED	10	mRNA	0.0	0.0	100.0	5.4^a^	± 0.365	42.5^a^	± 7.52
	7	protein	0.0	14.3	85.7	3.0^b^	± 0.398	9.1^b^	± 8.11
H11	11	mRNA	36.4	0.0	100.0	5.1^a^	± 0.396	70.9^a^	± 7.01
	13	protein	15.4	7.7	92.3	4.8^a^	± 0.353	33.7^b^	± 6.69
ZFX	12	mRNA	25.0	0	100.0	5.1^a^	± 0.375	79.7^a^	± 6.94
	9	protein	11.1	11.1	88.9	4.5^a^	± 0.386	43.5^b^	± 7.47

There was a decreased average number of alleles (3.0 ± 0.4) when targeting the POLLED locus using Cas9 protein (Fig. 1d; Table 2), as compared to Cas9 mRNA. There was no significant difference in the number of alleles for the other loci when comparing Cas9 mRNA or protein. However, there was a significant increase in the number of alleles when comparing pooled samples of embryos injected 18hpi with guides alongside Cas9 mRNA (5.23 ± 0.268), as compared to protein (4.23 ± 0.268) (ANOVA, *P* < 0.05). In addition, there was a significant increase in the percentage of wild type alleles present when injecting Cas9 mRNA compared to Cas9 protein for each of the three loci (42.5% vs. 9.1%, 70.9% vs. 33.7% and 79.7% vs. 43.5%, for POLLED , H11 and ZFX, respectively; *P* < 0.05).

A total of 24 potential off-target sites were predicted across 11 bovine chromosomes (1, 4, 7, 8, 10, 12, 14, 18, 21, 27 and X) (Supplementary Table S7) for the three loci. The 24 predicted off-target sites were PCR amplified, barcoded and sequenced using an Illumina MiSeq sequencer for each of the 69 samples (Supplementary Table S3). HTStream processed reads were aligned to the 24 predicted sites with 10,399,614 reads mapped with coverage ranging from 1X to 112X per sample per site (Supplementary Table S7). Genetic variation was found throughout the samples in each of the 24 predicted off-target sites with almost no indels present at the predicted off-target cut site with the exception of two targets. A 12 bp deletion 26 bp downstream from a predicted off-target cut site for the H11 gRNA targeting chr1: 7454978 was detected in 69,434 reads (6.8%) (Supplemental Table S7). Additionally, 2397 reads (0.51%) contained a 3 bp deletion 11 bp downstream from the predicted off-target cut site of the ZFX gRNA target chr21: 28506796 (Supplemental Table S7).

## Discussion

The ability to efficiently generate non-mosaic, homozygous founder animals is important for the production of genome edited livestock. The use of the CRISPR/Cas9 system has been reported across many livestock species^3^, but few reports have characterized its use in bovine embryos. In this study, using the CRISPR/Cas9 system, we identified gRNAs that resulted in high rates of mutation at target locations in two autosomes and the X chromosome in bovine embryos with an overall high efficiency (81–90%; Table 1). Significant differences were observed in gRNA efficiency within a locus, but not between loci. It has been demonstrated that microinjection itself does not have a significant impact on the development of bovine embryos^4^, but we found that microinjection of editing reagents in zygotes reduced development to the blastocyst stage compared to non-injected controls as the mutation efficiency of a given gRNA increased (Supplementary Table S2). However, no difference was observed in the number of embryos that reached the blastocyst stage when comparing embryos injected with Cas9 mRNA or protein (16.2% vs. 16.4%; Fig. 1a). This finding was important because we observed a significantly higher mutation rate in blastocysts when injecting Cas9 protein compared to Cas9 mRNA (84.2% and 68.5%, respectively; Fig. 1b). This difference is likely due to the immediate availability of the gRNA/Cas9 ribonucleoprotein (RNP) complex to induce mutation in the embryo. When Cas9 mRNA is injected, there is a delay in genome editing as Cas9 mRNA must be translated into protein before it can combine with the gRNA to induce a DSB^5^.

Mosaicism, the presence of more than two alleles in an individual, is a common problem in livestock genome editing^6^, with a high rate of embryos resulting in multiple alleles (Table 3). Studies utilizing transcription activator-like effector nucleases (TALENs) have demonstrated lower mosaicism rates than we observed here; however, the proportion of edited embryos tends to be lower as well^7,8^. A study employing a zinc finger nuclease (ZFN) in bovine embryos demonstrated both high embryo editing efficiency and mosaicism rates as compared to those found in TALEN edited embryos^9^. However, the prevalence of mosaicism was reduced when injecting embryos at 8hpi compared to 18hpi, before S-phase had occurred^9^. While we were able to induce mutations in embryos at a high rate, we also observed a high level of mosaicism when injecting 18hpi. Many studies of editing in livestock zygotes similarly report high levels of mosaicism when utilizing CRISPR/Cas9 (Table 3). Many of these studies characterized mosaicism by sequencing the PCR amplicon of the genomic regions flanking the gRNA target sequence and then decomposing the resulting chromatogram data with the TIDE bioinformatics package^10^. Although this approach is cost-effective and rapid, next generation sequencing of the PCR products allows for a more accurate characterization of the different alleles that are present in a mosaic individual, and their relative abundance^11^. However, this approach does present some concern with PacBio sequencing being highly error prone in regards to indels and SNPs. However, given the short sequences of the target amplicons, we were able to generate circular consensus sequencing (CCS) reads, increasing the confidence in the accuracy of the alleles that were being called^12^.

**Table 3:** Published results of genome editing targeting the NHEJ pathway in livestock zygotes, and rates of mosaicism (where available). Modified from Mclean et al^3^.

Nuclease^a^	Reagent^b^	Animal	Delivery Method^c^	Delivery time (post IVF)/h^d^	Target locus	Edited embryos %	Mosaic embryos %^e^	Edited offspring	Mosaic offspring	References
TALE	mRNA	Bovine	CI	19	*ACAN* or *GDF8*	2–50	20	–	–	^7^
TALE	mRNA	Bovine	CI	24	*GDF8*	31–57	ND	3/4	1/3	^8^
TALE	mRNA	Ovine	CI	24	*GDF8*	ND	ND	1/9	0/1	^8^
ZF	Plasmid	Bovine	CI	8	*LGB*	71	100	–	–	^9^
ZF	Plasmid	Bovine	CI	18	*LGB*	83	100	–	–	^9^
ZF	mRNA	Bovine	CI	8	*LGB*	70	75	–	–	^9^
ZF	mRNA	Bovine	CI	18	*LGB*	29	ND	–	–	^9^
Cas9	Plasmid	Porcine	CI	17	*GGTA1*	ND	ND	11/12	4/11	^43^
Cas9	mRNA	Ovine	CI	0	*PDX1*	67	38	2/4	2/2	^11^
Cas9	mRNA	Ovine	CI	6	*PDX1*	60	67	–	–	^11^
Cas9	mRNA	Ovine	CI	14–15	*BMPR–IB*	38	86	–	–	^44^
Cas9	mRNA	Ovine	CI	22	*MSTN*	50	80	10/22	4/10	^45^
Cas9	mRNA	Porcine	CI	3 (PA)	*Tet1*	94	30	–	–	^46^
Cas9	mRNA	Porcine	CI	8 (PA)	*Tet1*	100	33	–	–	^46^
Cas9	mRNA	Porcine	CI	18 (PA)	*Tet1*	83	100	–	–	^46^
Cas9	mRNA	Porcine	CI	?	*Npc1l1*	88	ND	11/11	9/11	^47^
Cas9	RNP	Bovine	CI	10 (IVF), 1 (PA)	*POU5F1*	86	34	–	–	^48^
Cas9	RNP	Bovine	E	10	*MSTN*	27–67	75–100	–	–	^49^
Cas9	RNP	Bovine	E	15	*MSTN*	19–67	92–100	–	–	^49^
Cas9	RNP	Porcine	CI	0	*GalT*	21	100	–	–	^50^
Cas9	RNP	Porcine	CI	0 + 6	*GalT*	23	100	–	–	^50^
Cas9	RNP	Porcine	CI	6	*GalT*	28–61	82–100	–	–	^50^
Cas9	RNP	Porcine	E	12	*TP53*	73–100	30–55	6/9	5/6	^51^
Cas9	mRNA	Bovine	CI	0	*PAEP* and *CSN2*	88	30	–	–	^14^
Cas9	RNP	Bovine	CI	0		87	30	–	–	^14^
Cas9	RNP	Bovine	CI	10		83	35	–	–	^14^
Cas9	mRNA	Bovine	CI	20		84	100	–	–	^14^
Cas9	RNP	Bovine	CI	20		83	100	–	–	^14^
Cas9	mRNA	Bovine	CI	18	POLLED	73	100	–	–	This study
Cas9	RNP	Bovine	CI	18	POLLED	86	86	–	–	This study
Cas9	mRNA	Bovine	CI	18	H11	70	100	–	–	This study
Cas9	RNP	Bovine	CI	18	H11	90	92	–	–	This study
Cas9	mRNA	Bovine	CI	18	ZFX	67	100	–	–	This study
Cas9	RNP	Bovine	CI	18	ZFX	81	89	–	–	This study

^a^Transcription activator-like effector (TALE), zinc finger (ZF). ^b^Nuclease delivered as plasmid, mRNA, or ribonucleoprotein (RNP) complex. ^c^Cytoplasmic injection (CI) or electroporation (E). ^d^In vitro fertilization (IVF) or parthenogenetic activation (PA). ^e^normalized on the total number of edited embryos or not determined (ND).

In bovine embryos, DNA replication occurs approximately 12–14 h after fertilization^13^. When injecting at 18hpi, as is often done when using traditional *in-vitro* fertilization (IVF) protocols, most zygotes would be expected to have completed DNA replication^14^ and there would likely be more than two copies of each chromosome, thus more opportunities for multiple genomic edits to occur, resulting in mosaicism. Additionally, following cytoplasmic injection, the gRNA/Cas9 ribonucleoprotein (RNP) complex needs time to enter the nucleus, find its target and cleave the DNA. Furthermore, if injecting Cas9 mRNA, translation to Cas9 protein must also occur, further delaying the editing process, thus resulting in a higher rate of mosaicism. It has been suggested that injection of the CRISPR/Cas9 RNP prior to the S-phase of DNA replication could reduce mosaicism^1^.

One recent study with bovine embryos reported low rates (~ 30%) of mosaicism when introducing Cas9 RNA or protein into early stage zygotes (0 or 10hpi) prior to the S-phase of DNA replication^14^. In that study, the authors were targeting two genes simultaneously via microinjection of two gRNAs into either matured oocytes before IVF or into zygotes at various time points post IVF. Allele identification was first made by Sanger sequencing of an amplicon of the targeted region, and then by clonal sequencing of 10 colonies derived from the PCR product per embryo. PCR and cloning-based approaches can identify that a range of alleles exist but cannot accurately quantitate the abundance of each allelic species. The authors went on to employ next generation sequencing on 20 embryos per group to characterize the alleles in non-mosaic embryos. The authors considered embryos that contained biallelic mutations resulting in frame-disrupting alleles to be non-mosaic, regardless of the number of alleles.

In the current study, we employed next generation sequencing to quantitate the abundance of each allele. The fact that we observed multiple alleles occurring in only a small percentage of reads (< 25%) in many samples analyzed in this study (Fig. 2) suggests that editing continued in some subset of cells after the first cleavage division. Further, we considered an embryo containing more than one population of genetically distinct cells to be mosaic irrespective of whether the edit resulted in a missense or nonsense mutation. It is important to determine if founder animals are mosaic because mosaicism complicates the interpretation of the effect of a given genome alteration^6^, and subsequent breeding of mosaic founder animals to achieve non-mosaic animals can take years^15^. Additionally, mosaics do not fit easily into the proposed regulatory framework for genome edited food animals^16^.

Along with the level of mosaicism, one of the concerns raised with the generation of genome edited animals is the potential for off-target mutation events. Typically, online prediction tools are used to calculate the likelihood of off-target sites^17–19^. The top predicted sites can then be PCR amplified and the presence of a mutation determined by either next generation sequencing, TA cloning followed by Sanger sequencing, or mismatch cleavage assays followed by Sanger sequencing^20^. In this study, we used the targeted approach using online predictive tools to identify off-target sites rather than a genome-wide approach. Off-target cleavage can occur in the genome with three to five base pair mismatches in the PAM-distal sequence^17,21–23^. Cas9 specificity is determined by the seed region, or the 8 to 11-nt PAM-proximal sequence, making it the most vital part of the gRNA sequence^21,24^. In our gRNA design, we excluded all gRNAs with less than three mismatches across the off-target sequence. We determined this threshold based on previous studies showing reduced Cas9 activity in regions with at least three mismatches^25^.

In the 69 samples analyzed, there were two potential off-target mutations detected. One of these (H11) was in a region that had known annotated wild type 12 bp deletions (rs876383581 and rs521367917) around the potential cut-site. Additionally, 0.51% of total reads contained a 3 bp deletion 11 bp downstream from the predicted off-target cut site for the ZFX gRNA target chr21: 28,506,796 (Supplemental Table S7). This predicted site does not have any annotated variation. It is important to note that although this off-target location had three mismatches to the gRNA sequence, all three of the mismatches were located outside the seed region (8–11 bp upstream of the PAM sequence). This guide was designed using off-target prediction software and the Btau 4.6.1 bovine reference genome^26^, which was the only *Bos taurus* reference genome available with the online tool at the time. When the off-target prediction software was re-run for the off-target analysis, the most recent reference genome available was UMD 3.1.1^26^. Using the new reference genome, this locus on chromosome 21 was identified as having the requisite three mismatches, but there were no mismatches in the seed region, as specified by our guide design criteria. More recently, an improved reference bovine genome ARS-UCD1.2 was published^27^. Using the online tool with the updated reference genome resulted in the same predicted off-target sites as UMD 3.1.1.

One of the stated concerns with off-target mutation events is that if they occur in functional regions, such as coding sequences or regulatory regions, they could potentially be detrimental to the health or development of the resulting animal. Neither of these two off-target deletions were in a region of annotated function. As there were approximately 20 individual blastocysts included in these analyses, these deletions may also have been the result of naturally occurring polymorphic variation. A detailed sequence analysis of 2703 individuals from different breeds of cattle revealed a high level of genetic diversity including 84 million single-nucleotide polymorphisms (SNPs) and 2.5 million small insertion deletions^28^. Data like these are essential to put naturally occurring variation, like that seen at the H11 locus, in context. Various studies in humans^29,30^, monkeys^31^, and rodents^32,33^ suggest that the off-target frequency of Cas9-mediated mutagenesis does not differ from the de novo mutation rate.

Overall, we demonstrated efficient CRISPR/Cas9 genome editing across three different loci on three different chromosomes. We found that injecting zygotes with Cas9 protein results in a significantly higher mutation rate compared to Cas9 mRNA (82.2% vs 65.4%). In addition, zygotes injected with Cas9 protein displayed a significantly lower number of alleles compared to those injected with Cas9 mRNA (4.2 vs 5.2). Although off-target events did not appear to be an issue, the rate of mosaicism was still high, and further optimization needs to be done before this technique is feasible in a livestock production setting.

## Materials and methods

### Guide construction

Guides sequences were designed using the online tools sgRNA Scorer 2.0^34,35^ and Cas-OFFinder^36^ and targeting the POLLED locus on chromosome 1, a safe harbor locus (H11) on chromosome 17 and in the 3′ UTR of the Zinc-finger X-linked (*ZFX*) gene (ZFX) on the X-chromosome. Guides were selected with no less than three mismatches in the guide sequence for off-target sites using the UMD3.1.1 bovine reference genome^26^, and at least one mismatch in the seed region (8–11 bp upstream of the PAM sequence). Oligonucleotides were ordered from Eurofins USA (Louisville, KY) for the top four guides for construction of the gRNA and were used for in vitro transcription using the AmpliScribe T7-Flash Transcription kit (Lucigen, Palo Alto, CA) and purified using the MEGAclear Transcription Clean-Up kit (Thermo Fisher, Chicago, IL) as described by Vilarino et al^11^. Cleavage efficiency was tested using an in vitro cleavage assay by combining 60 ng of PCR amplified product, 100 ng of gRNA, 150 ng of Cas9 protein (PNA Bio, Inc., Newbury Park, CA), 1 μL of 10X BSA, 1 μL of NEB Buffer 3.1 and water bringing the total volume to 10 μL in a 0.2 μL tube and incubating at 37 °C for 1 h. The incubated product was then run on a 2% agarose gel with 5 μL of Sybr Gold at 100 V for 1 h and visualized using a ChemiDoc-ItTS2 Imager (UVP, LLC, Upland, CA).

### Embryo production

Bovine ovaries were collected from a local processing plant and transported to the laboratory at 35–37 °C in sterile saline. Cumulus-oocyte complexes (COCs) were aspirated from follicles and groups of 50 COCs were transferred to 4-well dishes containing 400 μL of maturation media^37^. COCs were incubated for 21–24 hr at 38.5 °C in a humidified 5% CO_2_ incubator. Approximately 25 oocytes per drop were fertilized in 60 μL drops of SOF-IVF^37^ with 1 × 10^6^ sperm per mL and incubated for 18 hr at 38.5 °C in a humidified 5% CO_2_ incubator. Presumptive zygotes were denuded by light vortex in SOF-HEPES medium^37^ for 5 min. 25 zygotes per drop were incubated in 50 μL drops of KSOM culture media (Zenith Biotech, Glendale, CA, USA) at 38.5 °C in a humidified atmosphere of 5% CO_2_, 5% O_2_, and 90% N_2_ for 7–8 days.

### Guide testing

Mutation rate for each guide was determined by laser-assisted cytoplasmic injection^4^ of in vitro fertilized embryos with 6pL of a solution containing 67 ng/μL of in vitro transcribed gRNA alongside 133 ng/μL of Cas9 mRNA or 167 ng/μL of Cas9 protein (PNA Bio, Inc., Newbury Park, CA) incubated at room temperature for 30 min prior to injection. Injected embryos were incubated for 7–8 days. Embryos that reached blastocyst stage were lysed in 10 μL of Epicenter DNA extraction buffer (Lucigen, Palo Alto, CA) using a SimpliAmp Thermal Cycler (Applied Biosystems, Foster City, California) at 65 °C for 6 min, 98 °C for 2 min and held at 4 °C. The target region was amplified by two rounds of the polymerase chain reaction (PCR) using primers developed using Primer3 (Supplementary Table S1)^38,39^. The first round of PCR was performed on a SimpliAmp Thermal Cycler (Applied Biosystems, Foster City, California) with 10 μL GoTAQ Green Master Mix (Promega Biosciences LLC, San Luis Obispo, CA), 0.4 μL of each primer at 10 mM and 9.2 μL of DNA in lysis buffer for 5 min at 95 °C, 35 cycles of 30 s at 95 °C, 30 s at anneal temp (Supplementary Table S1), and 30 s at 72 °C, followed by 5 min at 72 °C. The second round of PCR was run with 10 μL GoTAQ Green Master Mix (Promega Biosciences LLC, San Luis Obispo, CA), 4.2 μL of water, 0.4 μL of each primer at 10 mM and 5 μL of first round PCR for 3 min at 95 °C, 35 cycles of 30 s at 95 °C, 30 s at anneal temp (Supplementary Table S1), and 30 s at 72 °C, followed by 5 min at 72 °C. Products were visualized on a 1% agarose gel using a ChemiDoc-ItTS2 Imager (UVP, LLC, Upland, CA), purified using the QIAquick Gel Extraction Kit (Qiagen, Inc., Valencia, CA) and Sanger sequenced (GeneWiz, South Plainfield, NJ).

### Allelic variation and off-target analysis

Embryos that reached the blastocyst stage were lysed and underwent whole-genome amplification using the Repli-G Mini kit (Qiagen, Inc., Valencia, CA). To determine presumptive off-target sites, guide sequences were mapped against the bosTau8 bovine reference genome using the online tool Cas-OFFinder^36^. A total of 24 off-target sites were predicted using the online tool: eight off-target sites for the POLLED gRNA, eleven off-target sites for the H11 gRNA and five off-target sites for the ZFX gRNA (Supplementary Table S7). Whole-genome amplified samples were used for PCR amplification of cut-sites and presumptive off-target sites using a dual round PCR approach described above to barcode each sample with a reduction from 35 to 5 cycles in the first round of PCR. Primers were designed to amplify each region using Primer3^38,39^ with a 15 bp adapter sequence attached to the forward (AGATCTCTCGAGGTT) and reverse (GTAGTCGAATTCGTT) (Supplementary Information S1). The second round of PCR amplified off the adapters adding an independent barcode for each sample to identify reads for pooled sequencing (Supplementary Table S1).

PCR samples targeting the gRNA cut site underwent SMRTbell library preparation and were sequenced on a PacBio Sequel II sequencer by GENEWIZ, LLC (South Plainfield, NJ, USA). Consensus sequences were called, reads sorted by barcode and BAM converted to individual FASTQ files using SMRT Link v8.0.0.80529 (https://www.pacb.com/support/software-downloads/). Reads were aligned to each target site using BWA v0.7.16a^40^. SAM files were converted to BAM files, sorted and indexed using SAMtools v1.9^41^. Number and types of alleles were determined for each sample using CrispRVariants v1.12.0^42^.

Off-target PCR samples underwent library preparation using the Illumina TruSeq library kit and were sequenced (300 bp paired-end) on an Illumina MiSeq Next Generation Sequencer by the DNA Technologies and Expression Analysis Cores at the UC Davis Genome Center. Paired-end reads were processed and overlapped to form high quality single-end reads using HTStream Overlapper v1.1.0 (https://github.com/ibest/HTStream). Processed reads were aligned to each target site using BWA v0.7.16a^40^. SAM files were converted to BAM files, sorted and indexed using SAMtools v1.9^41^. Insertions and deletions were called using CrispRVariants v1.12.0^42^.

### Statistical analysis

Comparison between blastocyst development and mutation rates were evaluated using a logistic regression model with Cas9 form and gRNA modeled as fixed effects. To analyze the level of mosaicism, an ANOVA test was used to determine significance between number of alleles per sample and percent wild type when injecting alongside Cas9 mRNA or protein. Samples with only wild type alleles were removed from analysis. Differences were considered significant when *P* < 0.05.

## Supplementary information

Supplementary Information.

## Data Availability

Raw sequence reads from PacBio Sequel II and Illumina MiSeq sequencing are available in the NCBI Sequence Read Archive as BioProject PRJNA623431 and SRA accession number SRR11850065. Individual results for the blastocyst development and mutation rate from each replicate (~ 30 embryos) of control and microinjected embryos are available in Supplementary Table S8.
