# Supplementary material for: Evaluation of mutation rates, mosaicism and off target mutations when injecting Cas9 mRNA or protein for genome editing of bovine embryos
Source: Sci Rep. 2020 Dec 18;10:22309. doi: 10.1038/s41598-020-78264-8 (PMC7749171; doi:10.1038/s41598-020-78264-8)
Supplement: Supplementary file 1 — Supplementary Information. [file 41598_2020_78264_MOESM1_ESM.docx]

**Supplementary Data**

**Evaluation of Mutation Rates, Mosaicism and Off Target Mutations when injecting Cas9 mRNA or Protein for Genome Editing of Bovine Embryos**

Sadie L. Hennig^#1^, Joseph R. Owen^#1^, Jason C. Lin^1^, Amy E. Young^1^, Pablo J. Ross^1^, Alison L. Van Eenennaam^1^ and James D. Murray^1,2^

^1^Department of Animal Science, University of California – Davis, CA, United States

^2^Department of Population Health and Reproduction, University of California – Davis, CA, United States

^#^ Sadie L. Hennig and Joseph R. Owen contributed equally.

Corresponding Author email: alvaneenennaam@ucdavis.edu

**Supplementary Table S1.** Sequence of primers used for PCR amplification of the POLLED, H11, or ZFX target regions, predicted off-target regions and gRNA sequences.

|  | **Name** | **Sequence 5’- 3’** | **T_m_ (^o^C)** |
| --- | --- | --- | --- |
| On-Target primers | POLLEDgF | GAAGTGTGGCCGGTAGAAAA | 62.8 |
|  | POLLEDgR | CGCTCCTTCCAAAACAAAAA | 60.4 |
|  | H11gF | CCCCAGTGTTGTGCATGTAG | 62.4 |
|  | H11gR | GTGAATGCCACTGCTGTGTT | 60.4 |
|  | ZFXgF | AGCAGTGCTTCCAAACTTGAG | 60.6 |
|  | ZFXgR | GATGAGAGCTTATGTAACTGTTGG | 61.2 |
| Off-Target primers | POLLEDoff1F | CAACTTCCCAGCTGTCTGC | 59.0 |
|  | POLLEDoff1R | CCTTGTATGACGGCAACCTT | 59.0 |
|  | POLLEDoff2F | TTCACTGCTCAAGGAAATGC | 58.4 |
|  | POLLEDoff2R | AAGGCTATGAACTTGGGCTTT | 58.7 |
|  | POLLEDoff3F | TTAAGCTTGGGCGTCTGAGT | 59.0 |
|  | POLLEDoff3R | CATTTGGCTTTCGGCTACAC | 59.0 |
|  | POLLEDoff4F | GAGGCAGATTTTGGCTTCAG | 60.4 |
|  | POLLEDoff4R | GCCTCTGTCCACATGCTCTT | 62.4 |
|  | POLLEDoff5F | CAGAGTCGGACACGACTGAA | 62.4 |
|  | POLLEDoff5R | GCTGTGTCCTCCTAGGCTCA | 64.5 |
|  | POLLEDoff6F | AAGGTTGTGTTGCATGTTGG | 59.0 |
|  | POLLEDoff6R | AATTCCACTCCTCCAGAATCA | 59.0 |
|  | POLLEDoff7F | TCTGGCATCACAGCATTTGT | 58.4 |
|  | POLLEDoff7R | AAGATGCAAGAGACGCAGGT | 60.4 |
|  | POLLEDoff8F | TTGGCCATGGACCTATGATT | 59.0 |
|  | POLLEDoff8R | GGAGTGACATGGCACCTCATA | 59.0 |
|  | H11off1F | GGAACAAAGATCCCACATGC | 59.0 |
|  | H11off1R | GGCAGTCAAAACCCAAACAC | 59.0 |
|  | H11off2F | GAATTCTGGGGGCATTGAC | 60.2 |
|  | H11off2R | GAAGCCTAACCACCTCCACA | 62.4 |
|  | H11off3F | CTCAGCTGGGTAACATGCAA | 60.4 |
|  | H11off3R | GAGCAAATTGAGGTGGGTAA | 58.4 |
|  | H11off4F | AATAAACCCCCAATTTGGCTA | 56.7 |
|  | H11off4R | GGACTATCCCCTGGAGAAGG | 64.5 |
|  | H11off5F | AGCCAGAGCTACTTGCTGGT | 62.4 |
|  | H11off5R | AGGGTTCACTCTTGTTGGTG | 60.4 |
|  | H11off6F | TGAATGGATAAGCTCCCTGTG | 60.6 |
|  | H11off6R | GAATGGTCCAGTGGTTGTCC | 62.4 |
|  | H11off7F | GGCAGAGAGGGAGAGAGACA | 64.5 |
|  | H11off7R | TTGCCAGACATGAGAAGCAG | 60.4 |
|  | H11off8F | CATGTAAATTTGGGGGTTGT | 57.0 |
|  | H11off8R | CCTTCTAATTCTTGTCTGTTTGCTT | 57.0 |
|  | H11off9F | CCTTGCAGATCAGCTCACAA | 60.4 |
|  | H11off9R | AATGGCTTCTTCCCTCAGGA | 60.4 |
|  | H11off10F | GGCTTTTTGCTCTGCTGTTT | 58.4 |
|  | H11off10R | TCAGAGGACCAGATGATGGA | 60.4 |
|  | H11off11F | GCACCGGGAGTTAATGTGTAA | 60.6 |
|  | H11off11R | AAGGGACAAGGTGTGGACTG | 62.4 |
|  | ZFXoff1F | GCAGCACCCAGAGTATCTCC | 64.5 |
|  | ZFXoff1R | CCTGAGGTAGGGGGATTGTT | 62.4 |
|  | ZFXoff2F | CCCCACTCCAGTACTCTTGC | 64.5 |
|  | ZFXoff2R | TCCCGTGTTTTGTGTGATTT | 56.3 |
|  | ZFXoff3F | TCATCTGGGCTGTTCTGAAG | 60.4 |
|  | ZFXoff3R | AAGGTTCCTGCCTGCTTTTT | 58.4 |
|  | ZFXoff4F | AAGGAAGGGGATTTTCTCCA | 58.4 |
|  | ZFXoff4R | CACAGGGCTTTCTCCTTGAG | 62.4 |
|  | ZFXoff5F | CAGCAAACTTTTCAGTGAGCA | 58.7 |
|  | ZFXoff5R | TCCTCTCCTTTTTGGACATCA | 58.7 |
| Barcode Primers | BC1001F | CACATATCAGAGTGCGAGATCTCTCGAGGTT | 62.0 |
|  | BC1001R | CACATATCAGAGTGCGGTAGTCGAATTCGTT | 62.0 |
|  | BC1002F | ACACACAGACTGTGAGAGATCTCTCGAGGTT | 62.0 |
|  | BC1002R | ACACACAGACTGTGAGGTAGTCGAATTCGTT | 62.0 |
|  | BC1003F | ACACATCTCGTGAGAGAGATCTCTCGAGGTT | 62.0 |
|  | BC1003R | ACACATCTCGTGAGAGGTAGTCGAATTCGTT | 62.0 |
|  | BC1004F | CACGCACACACGCGCGAGATCTCTCGAGGTT | 62.0 |
|  | BC1004R | CACGCACACACGCGCGGTAGTCGAATTCGTT | 62.0 |
|  | BC1006F | CATATATATCAGCTGTAGATCTCTCGAGGTT | 62.0 |
|  | BC1006R | CATATATATCAGCTGTGTAGTCGAATTCGTT | 62.0 |
|  | BC1007F | TCTGTATCTCTATGTGAGATCTCTCGAGGTT | 62.0 |
|  | BC1007R | TCTGTATCTCTATGTGGTAGTCGAATTCGTT | 62.0 |
|  | BC1008F | ACAGTCGAGCGCTGCGAGATCTCTCGAGGTT | 62.0 |
|  | BC1008R | ACAGTCGAGCGCTGCGGTAGTCGAATTCGTT | 62.0 |
|  | BC1009F | ACACACGCGAGACAGAAGATCTCTCGAGGTT | 62.0 |
|  | BC1009R | ACACACGCGAGACAGAGTAGTCGAATTCGTT | 62.0 |
|  | BC1010F | ACGCGCTATCTCAGAGAGATCTCTCGAGGTT | 62.0 |
|  | BC1010R | ACGCGCTATCTCAGAGGTAGTCGAATTCGTT | 62.0 |
| Guide RNA | POLLEDg1 | GTCTATCCCAAAAGTGTGGG | - |
|  | POLLEDg2 | CCTGTGAAATGAAGAGTACG | - |
|  | POLLEDg3 | GATAGTTTTCTTGGTAGGC | - |
|  | H11g1 | TAGCCATAAGACTACCTAT | - |
|  | H11g2 | CTGGGGCAAAAGTCAACAGT | - |
|  | H11g3 | TGACTGGGAGGAGGAAGCCA | - |
|  | ZFXg1 | GCTAGTGGGCTAATGCCAGA | - |
|  | ZFXg2 | GCCGTCTCTCTATAGCTCAG | - |
|  | ZFXg3 | TCTTACAAGGGTGATAGTAC | - |

**Supplementary Table S2.** Mutation rate in embryos for each guide injected 18 hours post insemination alongside Cas9 protein analyzed using PCR and Sanger sequencing. Multiple guides were tested targeting each locus to obtain highest efficiency guide. Letters that differ in the same column are significantly different (P < 0.05). Each chromosome independently tested using a two-by-two χ^2^ test.

| **Allele** | **gRNA** | **Injected Embryos** | **Total Blastocysts (%)** | **Blastocysts Analyzed** | **Mutation Rate (%)** |
| --- | --- | --- | --- | --- | --- |
| POLLED | 1 | 47 | 15 (32^a^) | 13 | 0 (0)^a^ |
|  | 2 | 75 | 14 (19^b^) | 13 | 10 (77)^b^ |
|  | 3 | 90 | 25 (28^a^) | 25 | 2 (8)^a^ |
| H11 | 1 | 65 | 12 (18^b^) | 12 | 10 (83)^b^ |
|  | 2 | 45 | 13 (29^a^) | 13 | 5 (38)^a^ |
|  | 3 | 47 | 10 (21^b^) | 10 | 6 (60)^ab^ |
| ZFX | 1 | 75 | 22 (29^a^) | 19 | 1 (5)^a^ |
|  | 2 | 86 | 22 (26^a^) | 21 | 5 (24)^a^ |
|  | 3 | 104 | 18 (17^b^) | 18 | 14 (78)^b^ |

**Supplementary Table S3.** List of sequencing barcodes used for PacBio sequencing for embryos injected 18 hours post insemination with gRNAs targeting the POLLED, H11, and ZFX loci alongside Cas9 mRNA or protein and corresponding reads per sample following sorting by barcode. Red highlighted samples were removed from analysis due to insufficient read count.

| **Locus** | **Cas9** | **Sample** | **Forward Barcode** | **Reverse Barcode** | **Reads per Sample** |
| --- | --- | --- | --- | --- | --- |
| POLLED | mRNA | 1 | BC1001F | BC1001R | 3359 |
|  |  | 2 | BC1001F | BC1002R | 1049 |
|  |  | 3 | BC1001F | BC1003R | 1446 |
|  |  | 4 | BC1001F | BC1004R | 1075 |
|  |  | 5 | BC1001F | BC1006R | 1118 |
|  |  | 6 | BC1001F | BC1007R | 598 |
|  |  | 7 | BC1001F | BC1008R | 472 |
|  |  | 8 | BC1001F | BC1009R | 2632 |
|  |  | 9 | BC1001F | BC1010R | 2662 |
|  |  | 10 | BC1002F | BC1001R | 2236 |
|  | protein | 1 | BC1002F | BC1003R | 24 |
|  |  | 2 | BC1002F | BC1004R | 276 |
|  |  | 3 | BC1002F | BC1006R | 812 |
|  |  | 4 | BC1002F | BC1007R | 654 |
|  |  | 5 | BC1002F | BC1008R | 12 |
|  |  | 6 | BC1002F | BC1009R | 543 |
|  |  | 7 | BC1002F | BC1010R | 1622 |
|  |  | 8 | BC1003F | BC1001R | 1445 |
|  |  | 9 | BC1003F | BC1002R | 417 |
| H11 | mRNA | 1 | BC1003F | BC1004R | 3762 |
|  |  | 2 | BC1003F | BC1006R | 3910 |
|  |  | 3 | BC1003F | BC1007R | 1203 |
|  |  | 4 | BC1003F | BC1008R | 3111 |
|  |  | 5 | BC1003F | BC1009R | 3267 |
|  |  | 6 | BC1003F | BC1010R | 2745 |
|  |  | 7 | BC1004F | BC1001R | 7681 |
|  |  | 8 | BC1004F | BC1002R | 1624 |
|  |  | 9 | BC1004F | BC1003R | 1579 |
|  |  | 10 | BC1004F | BC1004R | 1552 |
|  |  | 11 | BC1004F | BC1006R | 1937 |
|  | protein | 1 | BC1004F | BC1008R | 37 |
|  |  | 2 | BC1004F | BC1009R | 1693 |
|  |  | 3 | BC1004F | BC1010R | 8 |
|  |  | 4 | BC1006F | BC1001R | 6795 |
|  |  | 5 | BC1006F | BC1002R | 1197 |
|  |  | 6 | BC1006F | BC1003R | 1567 |
|  |  | 7 | BC1006F | BC1004R | 1926 |
|  |  | 8 | BC1006F | BC1006R | 2045 |
| **Locus** | **Cas9** | **Sample** | **Forward Barcode** | **Reverse Barcode** | **Reads per Sample** |
| H11 | protein | 9 | BC1006F | BC1007R | 1108 |
|  |  | 10 | BC1006F | BC1008R | 1472 |
|  |  | 11 | BC1006F | BC1009R | 1213 |
|  |  | 12 | BC1006F | BC1010R | 1937 |
|  |  | 13 | BC1007F | BC1001R | 2163 |
|  |  | 14 | BC1007F | BC1002R | 1838 |
|  |  | 15 | BC1007F | BC1003R | 1500 |
| ZFX | mRNA | 1 | BC1007F | BC1007R | 1630 |
|  |  | 2 | BC1007F | BC1008R | 1603 |
|  |  | 3 | BC1007F | BC1009R | 3973 |
|  |  | 4 | BC1007F | BC1010R | 3531 |
|  |  | 5 | BC1008F | BC1001R | 4960 |
|  |  | 6 | BC1008F | BC1002R | 1720 |
|  |  | 7 | BC1008F | BC1003R | 1521 |
|  |  | 8 | BC1008F | BC1004R | 1530 |
|  |  | 9 | BC1008F | BC1006R | 1039 |
|  |  | 10 | BC1008F | BC1007R | 17 |
|  |  | 11 | BC1008F | BC1008R | 2037 |
|  |  | 12 | BC1008F | BC1009R | 1484 |
|  |  | 13 | BC1008F | BC1010R | 1614 |
|  | protein | 1 | BC1009F | BC1001R | 4954 |
|  |  | 2 | BC1009F | BC1002R | 1240 |
|  |  | 3 | BC1009F | BC1003R | 27 |
|  |  | 4 | BC1009F | BC1004R | 1564 |
|  |  | 5 | BC1009F | BC1006R | 1280 |
|  |  | 6 | BC1009F | BC1007R | 1324 |
|  |  | 7 | BC1009F | BC1008R | 2102 |
|  |  | 8 | BC1009F | BC1009R | 2304 |
|  |  | 9 | BC1009F | BC1010R | 74 |
|  |  | 10 | BC1010F | BC1001R | 4014 |
|  |  | 11 | BC1010F | BC1002R | 1812 |

**Supplementary Table S4.** Number of PacBio sequencing reads of PCR products from 69 blastocysts microinjected with Cas9 editing reagents targeting three loci (POLLED, H11, and ZFX) in the bovine genome, and the percentage of reads that were <700 bp read length, and additionally had a unique blastocyst sample identifying barcode.

| **Filtered By** | **Locus** | | | | **Passed** | | **Total** | | **Percent** |
| --- | --- | --- | --- | --- | --- | --- | --- | --- | --- |
| Read length | | Total | | 171,545 | | 236,518 | | 72.5 | |
| Barcode | | | POLLED | | 22,416 | | 26,460 | | 84.7 |
| Barcode | | | H11 | | 58,815 | | 78,305 | | 75.1 |
| Barcode | | | ZFX | | 47,236 | | 66,780 | | 70.7 |

**Supplementary Table S5.** Prevalence of different allele types from PacBio sequencing of targeted PCR products < 700 bp from 69 blastocysts microinjected with Cas9 editing reagents targeting three loci (POLLED, H11, and ZFX) in the bovine genome. Types of mutations = location relative to the cut site (3bp upstream of the PAM sequence): type of deletion; D = deletion, I = insertion. “Other” mutations indicate those with reads too few to report.

| **Locus** | **Total Number of Reads** | **Wild Type Alleles (%)** | **Type of Mutation** | | | | |
| --- | --- | --- | --- | --- | --- | --- | --- |
|  |  |  | **# of Reads (%)** | | | | |
| POLLED | 26460 | 12719 (48) | **-16:7D** | **-14:11D** | **-13:4D** | **-10:1I** | **Other** |
|  |  |  | 1672 (6) | 1751 (7) | 6356 (24) | 2250 (19) | 1712 (6) |
| H11 | 78305 | 59302 (76) | **-14:11D** | **-13:6D** | **-12:3D** | **-10:1D** | **-8:1D** |
|  |  |  | 3246 (4) | 3813 (5) | 4091 (5) | 5061 (6) | 2792 (4) |
| ZFX | 66780 | 47939 (71) | **-10:14D** | **-4:9D** | **-1:3D** | **1:1D** | **2:1I** |
|  |  |  | 4222 (6) | 2998 (4) | 3198 (5) | 2194 (3) | 6532 (10) |

**Supplementary Table S6.** Number of alleles and percentage of each corresponding allele per sample detected at the cut-site of Cas9 mRNA or protein injected embryos. WT = percentage of reads that were wild type sequence. Alleles 1-5 are percent reads with each of the alleles found in the samples. Bold samples contained no wild type sequence. n/a = not applicable; genotypic sex was only determined for samples targeting the X chromosome.

| **Locus** | **Cas9** | **Sample** | **Sex** | **# of alleles** | **% of Reads for Each Allele** | | | | | | |
| --- | --- | --- | --- | --- | --- | --- | --- | --- | --- | --- | --- |
|  |  |  |  |  | **WT** | **Allele 1** | **Allele 2** | **Allele 3** | **Allele 4** | **Allele 5** |  |
| POLLED | mRNA | 1 | n/a | 5 | 57 | 22 | 10 | 8 | 3 | - |  |
|  |  | 2 | n/a | 5 | 76 | 11 | 7 | 4 | 2 | - |  |
|  |  | 3 | n/a | 6 | 37 | 44 | 9 | 6 | 4 | 3 |  |
|  |  | 4 | n/a | 5 | 9 | 31 | 28 | 25 | 8 | - |  |
|  |  | 5 | n/a | 6 | 46 | 22 | 20 | 6 | 6 | 4 |  |
|  |  | 6 | n/a | 5 | 67 | 17 | 11 | 3 | 2 | - |  |
|  |  | 7 | n/a | 4 | 9 | 75 | 13 | 2 | - | - |  |
|  |  | 8 | n/a | 6 | 20 | 43 | 27 | 7 | 4 | 3 |  |
|  |  | 9 | n/a | 6 | 53 | 23 | 13 | 7 | 4 | 3 |  |
|  |  | 10 | n/a | 6 | 39 | 21 | 21 | 15 | 5 | 4 |  |
|  | protein | 1 | n/a | 4 | 10 | 43 | 28 | 19 | - | - |  |
|  |  | 2 | n/a | 3 | 13 | 63 | 24 | - | - | - |  |
|  |  | 3 | n/a | 3 | 7 | 87 | 6 | - | - | - |  |
|  |  | 4 | n/a | 3 | 10 | 76 | 14 | - | - | - |  |
|  |  | **5** | n/a | **2** | **-** | **81** | **19** | **-** | **-** | **-** |  |
|  |  | 6 | n/a | 5 | 20 | 37 | 25 | 16 | 2 | - |  |
|  |  | **7** | n/a | **1** | **-** | **100** | **-** | **-** | **-** | **-** |  |
| H11 | mRNA | 1 | n/a | 6 | 54 | 34 | 4 | 3 | 3 | 3 |  |
|  |  | 2 | n/a | 6 | 21 | 44 | 20 | 6 | 5 | 4 |  |
|  |  | 3 | n/a | 5 | 14 | 65 | 17 | 2 | 2 | - |  |
|  |  | 4 | n/a | 6 | 46 | 27 | 14 | 7 | 3 | 3 |  |
|  |  | 5 | n/a | 5 | 72 | 16 | 6 | 4 | 2 | - |  |
|  |  | 6 | n/a | 4 | 84 | 13 | 2 | 1 | - | - |  |
|  |  | 7 | n/a | 4 | 93 | 4 | 2 | 1 | - | - |  |
|  |  | 8 | n/a | 1 | 100 | - | - | - | - | - |  |
| **Locus** | **Cas9** | **Sample** | **Sex** | **# of alleles** | **% of Reads for Each Allele** | | | | | |  |
|  |  |  |  |  | **WT** | **Allele 1** | **Allele 2** | **Allele 3** | **Allele 4** | **Allele 5** |  |
| H11 | mRNA | 9 | n/a | 1 | 100 | - | - | - | - | - |  |
|  |  | 10 | n/a | 1 | 100 | - | - | - | - | - |  |
|  |  | 11 | n/a | 1 | 100 | - | - | - | - | - |  |
|  | protein | 1 | n/a | 1 | 100 | - | - | - | - | - |  |
|  |  | 2 | n/a | 6 | 53 | 12 | 10 | 10 | 9 | 6 |  |
|  |  | 3 | n/a | 6 | 10 | 50 | 13 | 13 | 8 | 6 |  |
|  |  | 4 | n/a | 6 | 14 | 53 | 17 | 7 | 7 | 3 |  |
|  |  | 5 | n/a | 1 | 100 | - | - | - | - | - |  |
|  |  | 6 | n/a | 6 | 18 | 48 | 25 | 4 | 3 | 2 |  |
|  |  | 7 | n/a | 4 | 8 | 54 | 29 | 9 | - | - |  |
|  |  | 8 | n/a | 4 | 8 | 59 | 28 | 5 | - | - |  |
|  |  | **9** | n/a | **1** | **-** | **100** | **-** | **-** | **-** | **-** |  |
|  |  | 10 | n/a | 5 | 11 | 67 | 10 | 8 | 4 | - |  |
|  |  | 11 | n/a | 6 | 10 | 30 | 25 | 13 | 12 | 9 |  |
|  |  | 12 | n/a | 5 | 4 | 42 | 38 | 14 | 3 | - |  |
|  |  | 13 | n/a | 4 | 16 | 43 | 31 | 10 | - | - |  |
| ZFX | mRNA | 1 | female | 5 | 9 | 75 | 6 | 6 | 4 | - |  |
|  |  | 2 | male | 6 | 51 | 43 | 2 | 1 | 1 | 1 |  |
|  |  | 3 | female | 5 | 76 | 9 | 9 | 4 | 3 | - |  |
|  |  | 4 | female | 3 | 97 | 2 | 1 | - | - | - |  |
|  |  | 5 | male | 6 | 85 | 5 | 4 | 4 | 1 | 1 |  |
|  |  | 6 | female | 6 | 54 | 16 | 15 | 8 | 3 | 3 |  |
|  |  | 7 | male | 1 | 100 | - | - | - | - | - |  |
|  |  | 8 | female | 3 | 92 | 4 | 4 | - | - | - |  |
|  |  | 9 | female | 6 | 85 | 7 | 3 | 3 | 2 | 2 |  |
|  |  | 10 | female | 6 | 89 | 4 | 3 | 2 | 2 | 1 |  |
|  |  | 11 | female | 1 | 100 | - | - | - | - | - |  |
|  |  | 12 | male | 1 | 100 | - | - | - | - | - |  |
|  | protein | 1 | male | 5 | 65 | 12 | 8 | 7 | 7 | - |  |
| **Locus** | **Cas9** | **Sample** | **Sex** | **# of alleles** | **% of Reads for Each Allele** | | | | | |  |
|  |  |  |  |  | **WT** | **Allele 1** | **Allele 2** | **Allele 3** | **Allele 4** | **Allele 5** |  |
| ZFX | protein | 2 | male | 6 | 54 | 37 | 3 | 2 | 2 | 2 |  |
|  |  | 3 | female | 1 | 100 | - | - | - | - | - |  |
|  |  | 4 | female | 4 | 42 | 44 | 8 | 6 | - | - |  |
|  |  | 5 | male | 6 | 26 | 26 | 23 | 18 | 5 | 2 |  |
|  |  | **6** | male | **1** | **-** | **100** | **-** | **-** | **-** | **-** |  |
|  |  | 7 | male | 3 | 67 | 30 | 3 | - | - | - |  |
|  |  | 8 | female | 6 | 20 | 36 | 20 | 16 | 6 | 3 |  |
|  |  | 9 | female | 4 | 15 | 45 | 36 | 4 | - | - |  |

**Supplementary Table S7.** Predicted off-target sites for each of the three guides targeting the POLLED, H11, or ZFX locus. DNA = sequence of off-target site (lower case bases are mismatches). Position is relative to the start of the bosTau8 reference genome. Total reads aligned = number of reads mapped to the off-target sequence from overlapped MiSeq data. Coverage = reads per sample per target.

| **Target Locus** | **Off-Target Chromosome** | **DNA** | **Position** | **Direction** | **Total Reads Aligned** | **Coverage** |
| --- | --- | --- | --- | --- | --- | --- |
| POLLED | 2 | CaTGTGAAtTGAAGAGTACc | 17859417 | + | 301211 | 35X |
|  | 10 | CCTcTGgAATGAAGAGTACc | 23332597 | - | 94118 | 11X |
|  | 12 | CCTGTGAAATGActAGTACa | 57833284 | + | 256177 | 30X |
|  | 14 | CCTcTGAAATGAAGAGaACc | 83079285 | + | 85603 | 10X |
|  | 18 | CCTGaGAAATGAAGAGgAtG | 34058298 | - | 54877 | 6X |
|  | 18 | CtTGTGcAAaGAAGAGTACG | 46423386 | - | 301211 | 35X |
|  | X | CCTGTGAgATGAtGAtTACG | 31206746 | + | 430923 | 51X |
|  | X | gCTGTGAAATGAAGAGgAtG | 129076601 | + | 713874 | 84X |
| H11 | 1 | TAGCCATAAGcaTACCaAT | 3616887 | + | 21658 | 2X |
|  | 1 | TAGCCATAAGtCaACaTAT | 7454978 | + | 1022399 | 97X |
|  | 1 | TAGCCAcAAGtCTACaTAT | 12203491 | - | 878840 | 84X |
|  | 1 | gAGaCATAAGACTACCcAT | 111862587 | + | 226674 | 22X |
|  | 4 | TAGCCATAAGAaTtCCTAa | 102992457 | - | 249056 | 24X |
|  | 7 | TAGCaATAAGAgTACCTAa | 8578648 | - | 162124 | 15X |
|  | 7 | TAGtCATAAttCTACCTAT | 75383424 | - | 12118 | 1X |
|  | 7 | aAGCCATAcaACTACCTAT | 75200649 | - | 265923 | 25X |
|  | 8 | TAGCCATcAGACTACCaAg | 62416292 | - | 717812 | 68X |
|  | 10 | TAGCCAaAAGACaACaTAT | 59699661 | + | 1104588 | 105X |
|  | X | TAGCaATAAGAgTAaCTAT | 125300735 | + | 1179081 | 112X |
| ZFX | 7 | TCTTAaAAGGGTGATAaTAt | 112332349 | + | 125193 | 13X |
|  | 12 | TCTTACAgaGaTGATAGTAC | 22046100 | - | 474201 | 47X |
|  | 21 | aaTTACAgGGGTGATAGTAC | 28506796 | - | 470743 | 47X |
|  | 21 | TCTTACAAGaGTcATAGTgC | 48414495 | - | 329628 | 33X |
|  | 27 | TCcTAgAAGGGTGATcGTAC | 8648733 | - | 924826 | 92X |

**Supplementary Table S8.** Results for development and mutation rate from each replicate of control embryos, and groups injected 18 hours post insemination with gRNAs targeting the POLLED, H11, and ZFX loci alongside Cas9 mRNA or protein

| **Locus** | **Cas9** | **# Embryos** | **# Blastocyst** | **% Blastocyst** | **# Blastocysts**  **evaluated** | **# Blastocysts**  **With mutation** | **% Mutation** |
| --- | --- | --- | --- | --- | --- | --- | --- |
| POLLED | mRNA | 30 | 7 | 23.3 | 7 | 5 | 71.4 |
| POLLED | mRNA | 29 | 5 | 17.2 | 5 | 4 | 80 |
| POLLED | mRNA | 26 | 4 | 15.4 | 5 | 3 | 60 |
| POLLED | mRNA | 29 | 6 | 20.7 | 5 | 4 | 80 |
| POLLED | protein | 26 | 4 | 15.4 | 3 | 3 | 100 |
| POLLED | protein | 27 | 5 | 18.5 | 4 | 3 | 75 |
| POLLED | protein | 27 | 3 | 11.1 | 2 | 2 | 100 |
| POLLED | protein | 28 | 5 | 17.9 | 4 | 3 | 75 |
| POLLED | protein | 26 | 6 | 23.1 | 5 | 4 | 80 |
| POLLED | protein | 27 | 4 | 14.8 | 3 | 2 | 66.7 |
| POLLED | protein | 26 | 4 | 15.4 | 3 | 3 | 100 |
| POLLED | protein | 26 | 5 | 19.2 | 4 | 3 | 75 |
| POLLED | protein | 27 | 5 | 18.5 | 4 | 3 | 75 |
| POLLED | protein | 26 | 4 | 15.4 | 3 | 3 | 100 |
| POLLED | protein | 26 | 5 | 19.2 | 4 | 4 | 100 |
| POLLED | protein | 24 | 3 | 12.5 | 3 | 3 | 100 |
| H11 | mRNA | 26 | 3 | 11.5 | 3 | 2 | 66.7 |
| H11 | mRNA | 27 | 5 | 18.5 | 4 | 3 | 75 |
| H11 | mRNA | 26 | 3 | 11.5 | 3 | 2 | 66.7 |
| H11 | mRNA | 27 | 4 | 14.8 | 4 | 3 | 75 |
| H11 | mRNA | 25 | 3 | 12 | 3 | 2 | 66.7 |
| H11 | mRNA | 30 | 6 | 20 | 6 | 4 | 66.7 |
| H11 | mRNA | 30 | 4 | 13.3 | 4 | 3 | 75 |
| H11 | protein | 28 | 5 | 17.9 | 5 | 5 | 100 |
| H11 | protein | 30 | 6 | 20 | 6 | 5 | 83.3 |
| H11 | protein | 28 | 4 | 14.3 | 4 | 4 | 100 |
| H11 | protein | 28 | 4 | 14.3 | 4 | 4 | 100 |
| H11 | protein | 30 | 6 | 20 | 6 | 5 | 83.3 |
| H11 | protein | 30 | 5 | 16.7 | 5 | 4 | 80 |
| H11 | protein | 30 | 4 | 13.3 | 4 | 3 | 75 |
| H11 | protein | 30 | 5 | 16.7 | 5 | 5 | 100 |
| ZFX | mRNA | 26 | 4 | 15.4 | 4 | 3 | 75 |
| ZFX | mRNA | 26 | 4 | 15.4 | 4 | 2 | 50 |
| ZFX | mRNA | 27 | 5 | 18.5 | 5 | 3 | 60 |
| ZFX | mRNA | 26 | 5 | 19.2 | 5 | 3 | 60 |
| ZFX | mRNA | 26 | 4 | 15.4 | 4 | 3 | 75 |
| ZFX | mRNA | 27 | 4 | 14.8 | 4 | 2 | 50 |
| ZFX | mRNA | 26 | 3 | 11.5 | 3 | 2 | 66.7 |
| ZFX | mRNA | 25 | 5 | 20 | 5 | 4 | 80 |
| ZFX | mRNA | 26 | 3 | 11.5 | 3 | 1 | 33.3 |
| ZFX | mRNA | 25 | 4 | 16 | 4 | 3 | 75 |
| ZFX | mRNA | 26 | 5 | 19.2 | 5 | 4 | 80 |
| ZFX | mRNA | 26 | 4 | 15.4 | 4 | 2 | 50 |
| ZFX | mRNA | 30 | 6 | 20 | 6 | 5 | 83.3 |
| ZFX | mRNA | 30 | 7 | 23.3 | 6 | 4 | 66.7 |
| ZFX | protein | 26 | 4 | 15.4 | 4 | 3 | 75 |
| ZFX | protein | 26 | 4 | 15.4 | 4 | 3 | 75 |
| ZFX | protein | 27 | 5 | 18.5 | 5 | 4 | 80 |
| ZFX | protein | 26 | 5 | 19.2 | 5 | 5 | 100 |
| ZFX | protein | 26 | 4 | 15.4 | 4 | 3 | 75 |
| ZFX | protein | 24 | 4 | 16.7 | 4 | 3 | 75 |
| ZFX | protein | 26 | 3 | 11.5 | 3 | 3 | 100 |
| ZFX | protein | 25 | 5 | 20 | 5 | 4 | 80 |
| ZFX | protein | 26 | 3 | 11.5 | 3 | 2 | 66.7 |
| ZFX | protein | 25 | 4 | 16 | 4 | 3 | 75 |
| ZFX | protein | 26 | 5 | 19.2 | 5 | 4 | 80 |
| ZFX | protein | 26 | 4 | 15.4 | 4 | 4 | 100 |
| ZFX | protein | 26 | 4 | 15.4 | 4 | 3 | 75 |
| ZFX | protein | 25 | 5 | 20 | 5 | 4 | 80 |
| ZFX | protein | 25 | 4 | 16 | 4 | 3 | 75 |
| ZFX | protein | 26 | 4 | 15.4 | 4 | 4 | 100 |
| ZFX | protein | 24 | 3 | 12.5 | 3 | 2 | 66.7 |
| ZFX | protein | 25 | 4 | 16 | 4 | 3 | 75 |
| ZFX | protein | 26 | 4 | 15.4 | 4 | 3 | 75 |
| ZFX | protein | 26 | 3 | 11.5 | 3 | 2 | 66.7 |
| ZFX | protein | 25 | 4 | 16 | 4 | 4 | 100 |
| ZFX | protein | 25 | 6 | 24 | 5 | 4 | 80 |
| control | - | 30 | 9 | 30 | - | - | - |
| control | - | 30 | 8 | 26.7 | - | - | - |
| control | - | 30 | 7 | 23.3 | - | - | - |
| control | - | 30 | 6 | 20 | - | - | - |
| control | - | 30 | 13 | 43.3 | - | - | - |
| control | - | 30 | 8 | 26.7 | - | - | - |
| control | - | 30 | 10 | 33.3 | - | - | - |
| control | - | 29 | 9 | 31 | - | - | - |
| control | - | 30 | 8 | 26.7 | - | - | - |
| control | - | 30 | 13 | 43.3 | - | - | - |
| control | - | 30 | 8 | 26.7 | - | - | - |
| control | - | 30 | 9 | 30 | - | - | - |
| control | - | 30 | 7 | 23.3 | - | - | - |
| control | - | 30 | 8 | 26.7 | - | - | - |
| control | - | 27 | 9 | 33.3 | - | - | - |
| control | - | 30 | 10 | 33.3 | - | - | - |
| control | - | 30 | 8 | 26.7 | - | - | - |
| control | - | 30 | 5 | 16.7 | - | - | - |
| control | - | 29 | 7 | 24.1 | - | - | - |
| control | - | 30 | 6 | 20 | - | - | - |
| control | - | 30 | 8 | 26.7 | - | - | - |
| control | - | 29 | 9 | 31 | - | - | - |
| control | - | 30 | 8 | 26.7 | - | - | - |
| control | - | 30 | 14 | 46.7 | - | - | - |
| control | - | 30 | 8 | 26.7 | - | - | - |
| control | - | 28 | 13 | 46.4 | - | - | - |
| control | - | 30 | 8 | 26.7 | - | - | - |
| control | - | 30 | 7 | 23.3 | - | - | - |

**
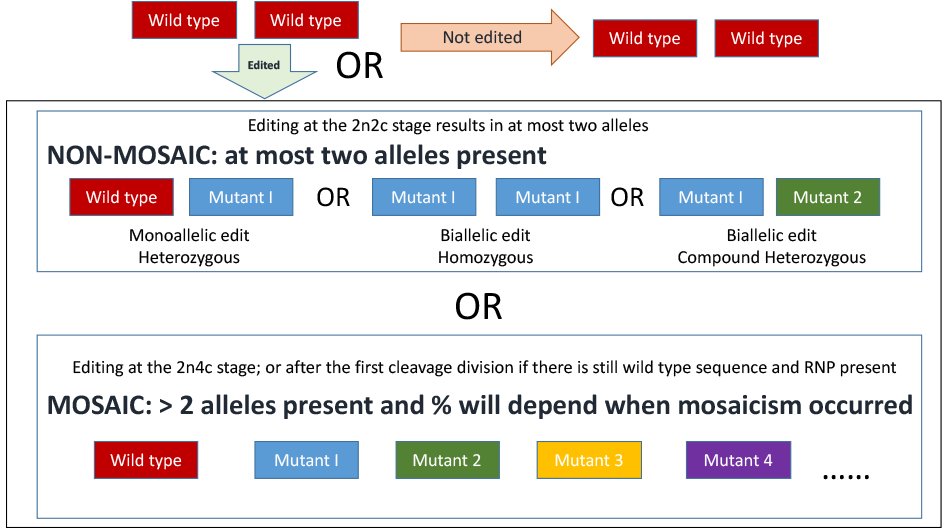
**

**Supplementary Figure S1.**  Schematic representation of possible outcomes from CRISPR-mediated mutation by cytoplasmic injection of an *in vitro* fertilized embryo 18 hours post insemination. 2n = number of homologous chromosomes, i.e. diploid. 2c/4c = number of copies of chromosomes either before DNA replication or after
